# Local Excision Without Radiation for High-Grade Soft-Tissue Sarcoma of the Extremity and Superficial Trunk

**DOI:** 10.1080/13577140020008075

**Published:** 2000-09

**Authors:** Lorna M. Weir, Anthony B. Vanbergeyk, Bassam A. Masri, Clive A. Grafton, Clive P. Duncan, Karen J. Goddard, Howard A. Joe

**Affiliations:** 1 Department of Radiation Oncology British Columbia Cancer Agency Vancouver Canada; 2 Department of Radiation Oncology British Columbia Cancer Agency Victoria Canada; 3 Department of Orthopaedics Faculty of Medicine University of British Columbia Vancouver Canada

## Abstract

*Purpose.* Limb-sparing surgery combined with radiation treatment has become the accepted treatment for patients with high-grade soft-tissue sarcoma. Adjuvant radiation was not routinely used at this institution for patients with clear margins after surgery.This retrospective review analyses the outcome of this group of patients.

*Patients and methods.* Patients studied were referred from 1984 to 1995, were over 16 years of age, were diagnosed with primary high-grade soft-tissue sarcoma of the extremity or superficial trunk, had clear margins after excision and did not receive radiation as a part of their initial treatment. A total of 46 patients were identified.

*Results.* At 5 years, the local control rate was 87%, disease-specific survival was 75% and overall survival was 68%. Of the 6 local recurrences, 3 were located in the buttock (from a total of 7 patients with primary tumours of the buttock), 3 had a primary size of ≥ 10 cm (from a total of 8 primary tumours of that size) and all were deep tumours.

*Discussion.* Our data, and those from other reports, suggest that in carefully selected patients appropriate surgery alone results in acceptable local control and survival, and that the morbidity of radiation can be avoided.


Sarcoma (2000) 4, 113–117

ORIGINAL ARTICLE

### Local excision without radiation for high-grade soft-tissue sarcoma of the extremity and superficial trunk

LORNA M. WEIR,[1] ANTHONY B. VANBERGEYK,[3] BASSAM A. MASRI,[3] CLIVE A. GRAFTON,[1] CLIVE P. DUNCAN,[3] KAREN J. GODDARD[1] & HOWARD A. JOE[2]

[1]*Department of Radiation Oncology, British Columbia Cancer Agency, Vancouver,* [2]*Department of Radiation Oncology, British Columbia Cancer Agency, Victoria and* [3]*Department of Orthopaedics, Faculty of Medicine, University of British Columbia, Vancouver, Canada*

**Abstract**

*Purpose*. Limb-sparing surgery combined with radiation treatment has become the accepted treatment for patients with high-grade soft-tissue sarcoma. Adjuvant radiation was not routinely used at this institution for patients with clear margins after surgery. This retrospective review analyses the outcome of this group of patients.

*Patients and methods*. Patients studied were referred from 1984 to 1995, were over 16 years of age, were diagnosed with primary high-grade soft-tissue sarcoma of the extremity or superficial trunk, had clear margins after excision and did not receive radiation as a part of their initial treatment. A total of 46 patients were identified.

*Results*. At 5 years, the local control rate was 87%, disease-specific survival was 75% and overall survival was 68%. Of the 6 local recurrences, 3 were located in the buttock (from a total of 7 patients with primary tumours of the buttock), 3 had a primary size of ≥ 10 cm (from a total of 8 primary tumours of that size) and all were deep tumours.

*Discussion*. Our data, and those from other reports, suggest that in carefully selected patients appropriate surgery alone results in acceptable local control and survival, and that the morbidity of radiation can be avoided.

**Key words**: *soft tissue sarcoma, local excision*

### Introduction

Soft tissue sarcomas (STSs) are rare neoplasms, accounting for approximately 1% of cancer cases diagnosed annually in the USA.[1] The treatment of the primary site continues to be refined with the development of newer surgical and radiation techniques. With surgical treatment alone, local recurrence (LR) rates are clearly related to the extent of surgery. After amputation or 'radical' local excision, LR rates of 2–13% are reported, compared with substantially higher LR rates with less radical surgery.[2,3] There are now many reports demonstrating acceptable LR rates after limb-sparing wide local excision (WLE) combined with pre- or post-operative radiation treatment.[3–8] Currently, most cancer treatment centres have adopted limb-sparing surgery and adjuvant radiation as the treatment of choice for STS, and amputation for primary treatment is recommended for very few patients.[9,10]

The use of adjuvant radiation, however, is associated with added morbidity that is not insignificant. Complications including fracture, oedema, contracture and poor wound healing have been reported.[11] Because of the morbidity associated with adjuvant radiation, it has not been used routinely at our institution in the management of primary STS. Our practice has been to employ limb-sparing WLE without radiation therapy for patients with negative margins following surgery. The purpose of this study is to review the outcome of 46 patients with high-grade STS of the extremity or superficial trunk treated in this fashion.

### Methods

All patients aged 16 or more that presented to the British Columbia Cancer Agency (BCCA) between 1 January 1984 and 31 December 1995 with high-grade STS of the extremity and superficial trunk were retrospectively reviewed. All patients had a pathological diagnosis of high-grade STS confirmed by central pathology review at the BCCA. The tumours were graded according to the system of Costa *et al.*[12] Patients were excluded from the analysis if they had metastases or recurrent disease at presentation, if they received adjuvant radiation treatment for the primary disease or if the definitive surgical

Correspondence to: Dr Lorna Weir, British Columbia Cancer Agency, 600 West 10th Ave, Vancouver, British Columbia V5Z 4E6, Canada. Tel: (604) 877-6000; Fax: (604) 877-0505; E-mail: lweir@bccancer.bc.ca

1357-714Xprint/1369-1643online/00/030113-05 © 2000 Taylor & Francis Ltd

*DOI: 10.1080/13577140020008075*

management consisted of anything other than limb-sparing WLE for extremity sites and WLE for trunk sites. In the latter part of the 1980s, adjuvant chemotherapy with doxorubicin and DTIC was used. Three patients in this study had post-operative chemotherapy as part of their initial management.

All patients were evaluated prior to surgical resection by a multidisciplinary team including Orthopaedics, Radiation Oncology, Medical Oncology, Radiology and Pathology. For those patients referred after an incomplete excision elsewhere, the definitive surgical resection was carried out by an experienced surgical oncologist at the tertiary care referral hospital affiliated to the BCCA. The tumour or tumour bed was resected *en bloc* with an attempt to resect at least 1 cm margin of normal tissue around the tumour in all planes. Any previous drain sites or biopsy scars were included in the resection. The resection specimen was reviewed by an experienced pathologist and by the multidisciplinary team after surgery. Patients were excluded if the definitive (i.e. final) surgical margin was positive. A margin was considered positive if tumour cells were seen within 1 mm of the inked resection margin. Patients who were not considered appropriate for surgery alone included the following: those who had a substantial amount of disease in a re-excision specimen after prior excisional biopsy; those with 'satellite' nodules; and patients whose surgery included an anticipated close margin (e.g. in the region of a neurovascular bundle or bone).

All patients were followed every 3–6 months for 5 years and then annually after definitive surgical resection. Local, regional and distant recurrences were recorded, as well as disease-specific and non-specific patient deaths. Overall survival, disease-specific survival and local and distant recurrence rates were calculated from the time of definitive surgical resection using the Kaplan–Meier statistical method.

### Results

A total of 145 patients aged 16 or older were identified through the computerized database of the BCCA as having high-grade STS of the extremity or superficial trunk, referred from January 1984 to December 1995 and having surgery alone as initial treatment. Exclusions were as follows: 79 cases with metastatic or locally recurrent disease at presentation; 10 cases having amputations; and 10 cases having their primary treatment delivered at another centre. This left a total of 46 cases, which are the subject of this review. Patient and tumour details are presented in Table 1. Only 9 patients had 'untouched' tumours prior to referral. The remaining 37 patients were referred after having either an incisional or an excisional biopsy without a pre-operative diagnosis or imaging.

Median follow-up was 4.4 years (range 3–14 years). Local control, disease-specific survival and overall survival rates are shown in Figs 1–3. The 5-year actuarial local control rate was 87%. The 5-year actuarial disease-specific survival and overall survival rates were 75% and 68%, respectively. A total of 15 patients died during the observation period, 12 of disease and 3 from unrelated causes.

**Table 1**. *Patient and tumour characteristics*

| | | |
|---|---|---|
| *Patients* | | |
| Age at diagnosis | Median | 63 |
| Gender | Male | 25 |
| | Female | 21 |
| *Tumours* | | |
| Size | <5 cm | 24 |
| | 5–9.9 cm | 8 |
| | ≥ 10 cm | 8 |
| | Unknown* | 6 |
| Location | Superficial | 17 |
| | Deep | 29 |
| | Trunk | 5 |
| | Buttock | 7 |
| | Proximal U/E | 7 |
| | Proximal L/E | 18 |
| | Distal U/E | 4 |
| | Distal L/E | 5 |
| Histology | MFH | 23 |
| | Synovial sarcoma | 3 |
| | Liposarcoma | 8 |
| | Fibrosarcoma | 3 |
| | Leiomyosarcoma | 6 |
| | Other | 3 |

MFH=maligant fibrous histiocytoma, U/E = upper extremity, L/E = lower extremity.
*No information on size available, tumour resected prior to referral.

The final surgical margin was stated to be negative, with no measurement given in 21 cases. In 4 cases, the re-excision specimen contained no tumour. The distance of the closest final margin was 1–4 mm in 11 cases, 5–10 mm in 7 cases and >10 mm in 3 cases.

Six patients had an LR and 11 patients had a distant recurrence. A breakdown of the recurrences is as follows: local only, 2; local and regional, 1; local followed by regional, 1; local/regional followed by distant, 1; distant only, 9; and distant followed by local, 1.

Of the 6 patients who had an LR, 3 had buttock primaries (from a total of 7 buttock primaries) and 3 had primary tumours ≥ 10 cm in size (from a total of 8 primaries of that size). Five of the six had biopsies prior to referral, and 1 patient had an 'untouched' tumour prior to referral. All LRs occurred in patients with deep tumours; no patient with a superficial tumour had an LR. In 2 cases the final margin was said to be negative, with no measurement given. For the remaining 4 cases, the closest final margins were 4 mm, 5 mm, 10 mm and 15 mm.

Patients with an LR had repeat excision and/or radiation therapy. Two of the six remained disease-free after a minimum follow-up of 3 years. The other 4 patients developed metastatic disease or had

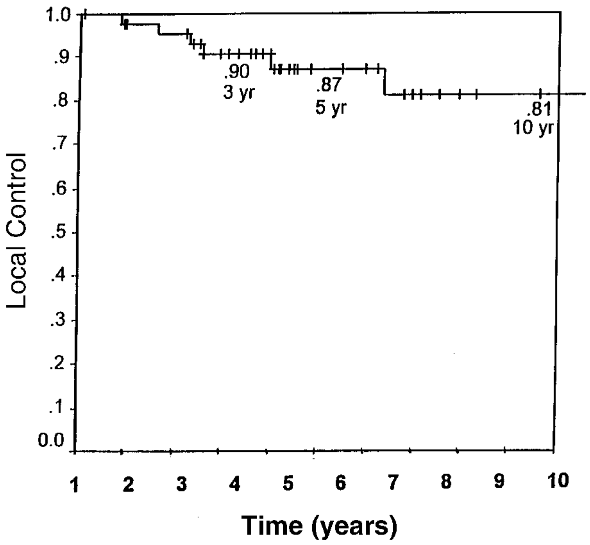

**Figure 1.** *Local control rates.*

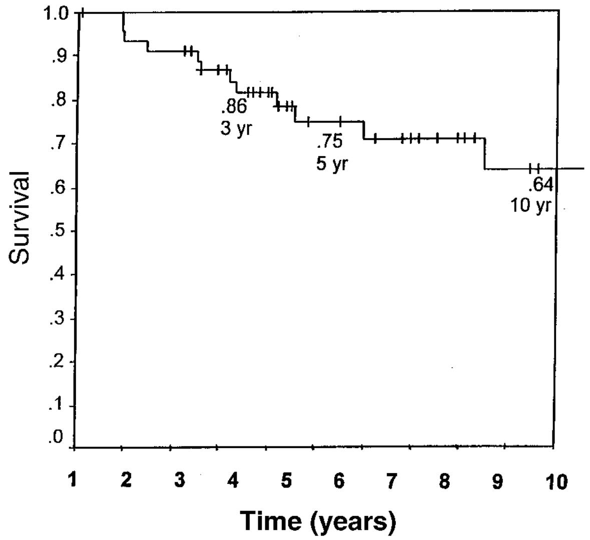

**Figure 2.** *Disease-specific survival rates.*

synchronous distant metastases and all eventually died of their disease. No patient had an amputation for LR. Of the 9 patients who had distant metastases alone, 1 had a resection of a pulmonary metastasis and remains disease-free after 10 years. The other 8 patients died of metastatic disease.

### Discussion

The literature is not replete with information about high-grade sarcoma treated with limb-salvage surgery without radiotherapy. In Scandinavia there has been a tradition of treating selected patients with sarcomas in intramuscular and subcutaneous sites with surgery alone and this experience has been reviewed by Rydholm.[13] The surgery for tumours in subcutaneous sites involved an *en bloc* resection which included the deep fascia, a 3–5 cm margin of surrounding tissue and usually the skin overlying the tumour. Most patients required skin grafting. After a median follow-up of 9 years, 4 of 59 high-grade tumours recurred locally. Patients who had had a previous biopsy or marginal excision at another centre were included in this group and did not have a higher recurrence rate.

Intramuscular tumours were treated with myectomy, with adjacent muscles resected if there was no fascial boundary between them. Only patients without a previous open biopsy were treated this way. Rydholm reported that 2 of 24 patients had an LR after a minimum follow-up of 3.5 years. Twenty of these cases were high-grade sarcomas. The subsequent clinical course of the patients with LR is not described, but he concluded that a local failure rate of 10% does not justify adjuvant radiation treatment.

Karakousis *et al.* reported on a group of 152 primary extremity STSs, treated in a variety of ways.[14] In this group there were 97 patients with high-grade tumours treated with wide excision, with or without chemotherapy, with an LR rate of 12%.

Gibbs *et al.* reviewed a group of 62 patients with subcutaneous extremity sarcomas, 35 of whom had

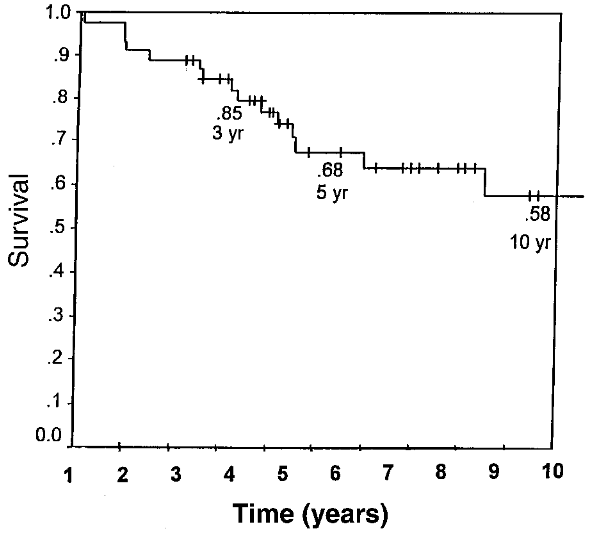

**Figure 3.** *Overall survival rates.*

wide excision alone.[15] Patients with better prognostic factors (lower grade, smaller size and wider margins) tended to be selected for surgery alone. There were no local recurrences in this group after a median follow-up of 56 months.

Two randomized studies comparing surgery alone with surgery and radiation have been published. Pisters *et al.* reported on the outcome of patients randomized to surgery alone versus surgery with brachytherapy, after a median follow-up of 76 months.[16] One hundred and sixty-four patients with extremity or superficial trunk sarcomas were randomized. After a gross total resection of the tumour, patients were randomized intra-operatively to receive brachytherapy or not. On definitive histological assessment, 29 patients were found to have positive margins (tumour within 1 mm of a margin). These patients were distributed evenly in both arms of the study. Thirty-four patients in each group also received postoperative chemotherapy. In the group with high-grade lesions ($N$=119), actuarial freedom from local recurrence at 5 years was 89% for brachytherapy and 66% for no brachytherapy ($p$=0.003). The benefit was confined to patients with high-grade tumours. There was no difference in disease-specific survival or freedom from distant metastases between the two arms.

Yang *et al.* published the results of a randomized study comparing surgery alone with surgery and external beam radiation in patients with extremity STS.[17] A total of 141 patients were randomized. Patients with high-grade tumours ($N$=91) were randomized to have limb-sparing surgery plus chemotherapy versus limb-sparing surgery plus concurrent chemotherapy and radiation. Patients with a limited positive margin were included. After a median follow-up of 9.6 years, the actuarial local failure rate in the chemotherapy alone arm was 22%. There were no LRs in the chemoradiation arm. There was no difference in the 10-year rate of distant metastases or overall survival between the two arms. Of the 9 patients with an LR, 4 had synchronous distant metastases and 3 died of metastatic sarcoma. Quality of life analysis showed that there was a persistent reduction in joint motion and a transient, but significant, increase in limb weakness and oedema in the patients who received radiation therapy. However, global quality of life and performance in activities of daily living were not different in the two groups. Because of the small number of local failures, no risk factors could be identified for LR (other than lack of radiation). The authors concluded that, while radiation did reduce the LR rate, no patients with widely negative margins treated with surgery alone had an LR. Therefore, for selected patients in whom the toxicity of radiation is expected to be high and the LR rate is expected to be low, surgery alone may be the treatment of choice.

We evaluated patients treated with limb-sparing surgery for extremity STS and WLE for superficial trunk STS. As a matter of policy, if the margins were clear (no tumour cells within 1 mm of the inked resection margin), adjuvant radiation was not routinely given. The distance of the final surgical margin varied from 1 mm to 20 mm. The LR rate was 13% at 5 years. The disease-specific and overall survival rates of 75% and 68%, respectively, were comparable to other reported series where adjuvant radiation was given routinely. Among the patients with an LR, tumours located in the buttock and tumours 10 cm or more in size were over-represented. These patients may not be suitable candidates for treatment with surgery alone. In addition, the LRs were seen only in patients with deep tumours.

In the present series, no patient had an amputation for LR. In Yang *et al.*'s study,[17] there were 6 patients in the no radiation group who had an LR without distant metastases. Two of these required an amputation. By way of comparison, Catton *et al.* reported that 7 of 25 patients required amputation for LR after initial treatment with limb-sparing surgery and radiation.[18] Stinson *et al.* reported that 3 of 145

patients required an amputation for treatment complications after limb-sparing surgery and radiation.[11] Even with these small numbers of patients, it does not appear that avoidance of radiation ultimately leads to a higher amputation rate because of LR.

These data confirm other reports that surgery alone is an acceptable treatment for carefully selected patients with high-grade STS of the extremity and superficial trunk. LR rates of 10–15% after surgery alone may not justify routine adjuvant radiation in these cases. The lowest recurrence rate is seen in the group with superficial tumours, even when an incisional or excisional biopsy was performed prior to referral. Deeply located tumours are more likely to recur with surgery alone, making this approach less appropriate. Strict adherence to oncological surgical principles, careful and thorough assessment of the pathological specimen and multidisciplinary care are all essential in selecting patients for this approach.

### References

1 Zahm SH, Fraumeni JFJr. The epidemiology of soft tissue sarcoma. *Semin Oncol* 1997;24:504–14.
2 Simon MA, Enneking WF. The management of soft tissue sarcomas of the extremities. *J Bone Joint Surg* 1976;58A:317–27.
3 Leibel SA, Tranbaugh RF, Wara WM, Beckstead JH, Bovill EG, Phillips TL. Soft tissue sarcomas of the extremities. *Cancer* 1982;50:1076–83.
4 Lindberg RD, Martin RG, Romsdahl MM, Barkley HT. Conservative surgery and postoperative radiotherapy in 300 patients with soft-tissue sarcomas. *Cancer* 1981;47:2391–7.
5 Suit HD, Mankin HJ, Wood WC, Gebhardt MC, Harmon DC, Rosenberg A *et al.* Treatment of the patient with stage Mo soft tissue sarcoma. *J Clin Oncol* 1988;6:854–62.
6 Fein DA, Lee WR, Lanciano RM, Corn BW, Herbert SH, Hanlon AL *et al.* Management of extremity soft tissue sarcomas with limb-sparing surgery and postoperative irradiation: do total dose, overall treatment time, and the surgery–radiotherapy interval impact on local control? *Int J Radiation Oncology Biol Phys* 1995;32:969–76.
7 Abbatucci JS, Boulier N, deRanieri J, Mandard AM, Tanguy A, Vernhes JC *et al.* Local control and survival in soft tissue sarcomas of the limbs, trunk walls and head and neck: a study of 113 cases. *Int J Radiation Oncology Biol Phys* 1986;12:579–86.
8 Barkley HT, Martin RG, Romsdahl MM, Lindberg R, Zagars GK. Treatment of soft tissue sarcomas by preoperative irradiation and conservative surgical resection. *Int J Radiation Oncology Biol Phys* 1988;14:693–9.
9 Williard WC, Hajdu SI, Casper ES, Brennan MF. Comparison of amputation with limb-sparing operations for adult soft tissue sarcoma of the extremity. *Ann Surg* 1992;215:269–75.
10 LeVay J, O'Sullivan B, Catton C, Bell R, Fornasier V, Cummings B *et al.* Outcome and prognostic factors in soft tissue sarcoma in the adult. *Int J Radiation Oncology Biol Phys* 1993;27:1091–9.
11 Stinson SF, DeLaney TF, Greenberg J, Yang JC, Lampert MH, Hicks JE *et al.* Acute and long-term effects on limb function of combined modality limb sparing therapy for extremity soft tissue sarcoma. *Int J Radiation Oncology Biol Phys* 1991;21:1493–9.
12 Costa J, Wesley RA, Glatstein E, Rosenberg SA. The grading of soft tissue sarcomas. Results of a clinicopathologic correlation in a series of 163 cases. *Cancer* 1984;53:530–41.
13 Rydholm A. Surgery without radiotherapy in soft tissue sarcoma. *Acta Orthop Scand* 1997;68 (Suppl. 273):117–9.
14 Karakousis CP, Proimakis C, Walsh DL. Primary soft tissue sarcoma of the extremities in adults. *Br J Surg* 1995;82:1208–12.
15 Gibbs CP, Peabody TD, Mundt AJ, Montag AG, Simon MA. Oncological outcomes of operative treatment of subcutaneous soft-tissue sarcomas of the extremities. *J Bone Joint Surg* 1997;79A:888–97.
16 Pisters PW, Harrison LB, Leung DH, Woodruff JM, Casper ES, Brennan MF. Long-term results of a prospective randomized trial of adjuvant brachytherapy in soft tissue sarcoma. *J Clin Oncol* 1996;14:859–68.
17 Yang JC, Chang AE, Baker AR, Sindelar WF, Danforth DN, Topalian SI *et al.* Randomized prospective study of the benefit of adjuvant radiation therapy in the treatment of soft tissue sarcomas of the extremity. *J Clin Oncol* 1998;16:197–203.
18 Catton C, Davis A, Bell R, O'Sullivan B, Fornasier V, Wunder J, McLean M. Soft tissue sarcoma of the extremity. Limb salvage after failure of combined conservative therapy. *Radioth Oncol* 1996;41:209–14.
